# Impact of *Leishmania* Infection on Host Macrophage Nuclear Physiology and Nucleopore Complex Integrity

**DOI:** 10.1371/journal.ppat.1004776

**Published:** 2015-03-31

**Authors:** Amandine Isnard, Jan G. Christian, Mohamed Kodiha, Ursula Stochaj, W. Robert McMaster, Martin Olivier

**Affiliations:** 1 Departments of Medicine and Microbiology & Immunology, The Research Institute of the McGill University Health Centre, McGill University, Montréal, Quebec, Canada; 2 Department of Physiology, McGill University, Montréal, Quebec, Canada; 3 Department of Medical Genetics, University of British Columbia, Vancouver, British Columbia, Canada; Imperial College London, UNITED KINGDOM

## Abstract

The protease GP63 is an important virulence factor of *Leishmania* parasites. We previously showed that GP63 reaches the perinuclear area of host macrophages and that it directly modifies nuclear translocation of the transcription factors NF-κB and AP-1. Here we describe for the first time, using molecular biology and in-depth proteomic analyses, that GP63 alters the host macrophage nuclear envelope, and impacts on nuclear processes. Our results suggest that GP63 does not appear to use a classical nuclear localization signal common between *Leishmania* species for import, but degrades nucleoporins, and is responsible for nuclear transport alterations. In the nucleoplasm, GP63 activity accounts for the degradation and mislocalization of proteins involved amongst others in gene expression and in translation. Collectively, our data indicates that *Leishmania* infection strongly affects nuclear physiology, suggesting that targeting of nuclear physiology may be a strategy beneficial for virulent *Leishmania* parasites.

## Author Summary

Unicellular parasites of the genus *Leishmania* are the causative agent of leishmaniasis, a disease affecting 12 million people worldwide, mainly in tropical and subtropical regions of the developing world. They have evolved strategies to circumvent cellular defense mechanisms favouring their survival. This includes the cleavage and activation of proteins and the subsequent block of signals within the host cells. In this study we discovered that a *Leishmania* virulence factor, GP63, is able to reach host cell nuclei and affect protein transport from and into the nucleus. Through the analysis of the protein content of nuclei after parasite infection we revealed that *Leishmania*, predominantly through the protein cleaving enzyme GP63, can alter several processes within the nucleus, amongst others mechanisms associated with gene expression and nucleic acid metabolism. Thus, we here introduce a novel strategy of how *Leishmania* parasites may overcome host cell defense and ensure their own survival.

## Introduction

The protozoan species *Leishmania major* (*L*. *major*) and *L*. *mexicana* are the causative agent of the cutaneous form of leishmaniasis, an ulcerative disease with ~1 million new cases reported worldwide annually. In mammalian hosts, *Leishmania* is an intracellular parasite that replicates predominantly within macrophages (MΦ). To survive, *Leishmania* suppresses microbicidal and immune functions of the MΦ, caused by alterations of signaling pathways [1]. A crucial molecule for the subversion of host signaling is the leishmanial surface metalloprotease GP63 [2].

In recent years GP63 was found to be a prerequisite not only for the activation of host protein tyrosine phosphatases (PTPs), such as SHP-1, PTP1B and TCPTP [3], but also for the alteration of transcription factor (TF) function through proteolytic cleavage [4]. Interestingly, GP63-mediated cleavage in the case of the TF AP-1 was shown to occur in the nucleus rather than the cytoplasm revealing that a parasite protease may enter the nucleus via an undefined mechanism [5].

In eukaryotic cells, the majority of nucleocytoplasmic exchange occurs through nuclear pore complexes (NPCs). Those channels are composed of nucleoporins (Nups) and enable passive transport of smaller molecules. Larger proteins usually require a nuclear localization sequence (NLS) and carrier proteins to be transported through the NPC. Apart from nuclear transport, NPC and Nups have also been implicated in other critical cellular processes such as cell differentiation, genome organization, and gene expression [6–8]. To date, a number of obligate intracellular pathogens (e.g. rhinovirus, poliovirus, HIV-1) have been shown to interfere with the nucleocytoplasmic transport machinery including the degradation of Nups and/or carrier proteins [9–11]. *Leishmania* parasites have been shown to affect host TF proteins and possibly their transport to the nucleus [4,5,12,13]. However, whether *Leishmania* parasites are able to further subvert host nuclear functions is unknown. Thus, the aim of this study was to elucidate the consequences of *Leishmania* infections on nuclear physiology with a special focus on the importance of GP63. We here show that GP63 targets the nuclear envelope (NE) after *Leishmania* infection of murine MΦs. As a result GP63 degrades Nups of the NPC. Thereby, translocation of the leishmanial protease to the NE appears to be independent of a putative classical NLS. Proteomic analysis of MΦ nuclei after infection reveals an impact of GP63 on critical proteins involved in nuclear transport, nucleic acid metabolism, and essential mRNA processes. Comparison of the proteomic data sets of *L*. *major* and *L*. *mexicana* infections shows that both species affect predominantly chromatin remodeling and transcriptional/translational regulation, likely through GP63. To our knowledge, this is the first in-depth proteomic analysis of macrophage nuclei after *Leishmania* infection revealing extensive alterations of nuclear physiology.

## Results

### GP63 Localizes at the Nuclear Envelope

We previously demonstrated that the *Leishmania* metalloprotease GP63 was able to cleave substrate proteins within the nucleus of host MΦs and can be localized in the perinuclear area of the nuclei [5].

**Fig 1:**
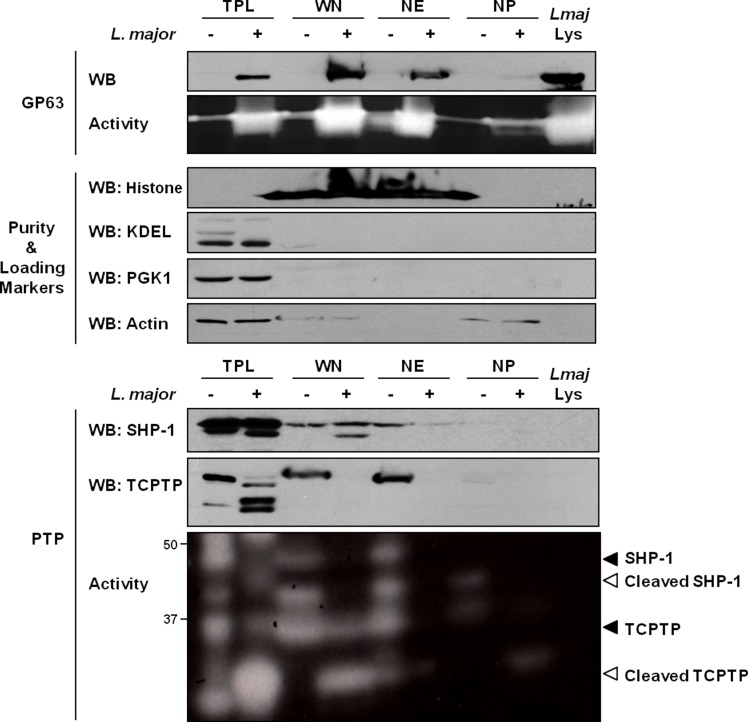
GP63 localizes at the nuclear envelope of host macrophages. Analysis of GP63 localization by immunoblot analysis. LM1 MΦs were infected as indicated with *L*. *major* for 2 hrs. Total protein lysate (TPL), whole nucleus (WN), nuclear envelope (NE), and nucleoplasm (NP) samples were generated. Parasite lysate (*Lmaj* Lys) was used as a control. Samples were analyzed by western blot, by gelatin zymography to test GP63 enzymatic activity, or by In Gel PTP assay to detect PTP enzymatic activity (active enzymes appeared as clear bands). An antibody against *Leishmania* GP63 was used to monitor its localization. Antibodies against SHP-1 and TCPTP were used to monitor their cleavage in presence of GP63. Histone, KDEL, PGK1 and Actin were used as controls.

In our experiments, we initially sought to confirm the previously observed distribution of GP63 within host cells after infection. In the conditions used, in accordance with the work of Contreras et al., cells were typically infected by 1–2 parasites and images of host cells after infection indicate a preference of GP63 to localize in the perinuclear region (S1 Fig). To investigate the nuclear localization of GP63 more accurately, we optimized a protocol to purify whole nuclei (WN) from MΦs and to fractionate the nuclear envelope (NE) from the nucleoplasm (NP). Our western blot analysis confirmed that *L*. *major* GP63 is indeed present in nuclear fraction (WN) of infected MΦ. In-depth analysis of GP63 localization within the nucleus revealed that the protease predominantly remains at the NE (Fig 1). This was supported by gelatin zymography, which showed strong GP63 activity at the NE. These results coincided with the finding that two GP63 targets, the PTPs SHP-1 and TCPTP, were primarily localized at the NE putting protease and substrates in close proximity (Fig 1).

### GP63-Dependent Cleavage of Nuclear Localized Substrates Is Independent of a Putative Classical NLS

Most nuclear proteins with a molecular mass of more than 40 kDa are transported into the nucleus through NPCs via the recognition and binding of a NLS by importins. The best-characterized transport signal is the classical NLS (cNLS) for nuclear protein import, which consists of either one (monopartite) or two (bipartite) stretches of basic amino acids [14].

**Fig 2:**
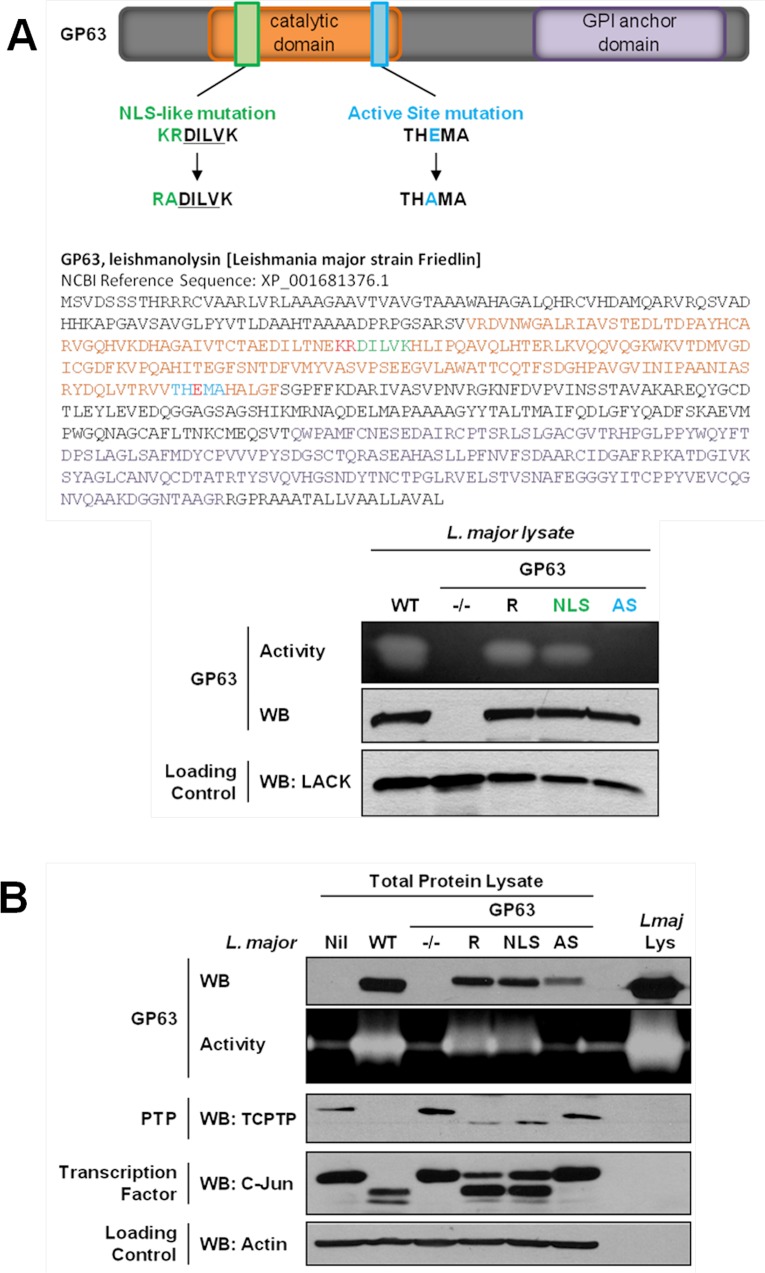
GP63-dependent cleavage of nuclear localized substrates is NLS sequence independent. (A) *L*. *major* GP63 mutants. (Top) Description of the mutations induced for *L*. *major* GP63^NLS^ and GP63^AS^ mutants. (Bottom) Parasite lysates of the different *L*. *major* species were analyzed by western blots and gelatin zymography to monitor GP63 presence and activity. The *Leishmania* parasite protein LACK was used as a loading control. (B) LM1 MΦ were infected or not (Nil = non-infected LM1) as indicated with *L*. *major* species (WT, GP63^-/-^, GP63^R^, GP63^NLS^, GP63^AS^) for 2 hrs, and cell lysates were subjected to total protein lysis. Parasite lysate (*Lmaj* Lys) was used as a control. Samples were analyzed either by western blot or by gelatin zymography. An antibody against *Leishmania* GP63 was used to monitor its presence. TCPTP was used to monitor the GP63-induced cleavage of PTPs. AP-1 C-Jun was used to monitor GP63-induced cleavage of TFs. Actin was used as a loading control.

We first searched for a cNLS in the GP63 sequence which is common to most *Leishmania* species. The PSORT II software did not identify a common cNLS [15]. However, we identified a motif very similar to a described consensus monopartite cNLS [16]. Mutants for this sequence (GP63^NLS^) were generated by site-directed mutagenesis. As the cNLS-like sequence was localized in the GP63 catalytic domain, we also generated a GP63 mutant for the active site (GP63^AS^, E265D) to discriminate between effects on the cNLS—and possibly transport—and effects on protease activity [17] (Fig 2A). *L*. *major* GP63 knockout mutant parasites (GP63^-/-^) and *L*. *major* GP63^-/-^ parasites complemented by transfection of the *L*. *major* GP63 gene 1 (GP63 Rescued; GP63^R^) [18] were used as controls for the new parasite strains (Fig 2 **)**.

Examination of GP63 activity demonstrated that GP63 was not functional in GP63^AS^ parasites while it was unaffected in GP63^NLS^ parasites. Hence, we concluded that the mutation in the cNLS-like sequence did not alter GP63 proteolytic activity (Fig 2A). In order to investigate whether GP63 utilized this putative cNLS-like sequence to target the NE enabling substrate cleavage events, we infected MΦs with different *L*. *major* species (WT, GP63^NLS^, GP63^AS^, GP63^-/-^ and GP63^R^) or not (Nil = non-infected LM1), and monitored GP63 as well as GP63-mediated cleavage of TCPTP and c-Jun. Both substrate cleavage events were previously reported to take place in the nucleus or nuclear fractions, respectively [3,5]. The infection experiments showed that all GP63-expressing parasite strains but *L*. *major* GP63^AS^ were able to cleave the substrates analyzed (Fig 2B). GP63^R^ and GP63^NLS^ parasites had very similar GP63 expression and substrate cleavage patterns, probably due to the fact that only one GP63 gene copy was rescued [18]. The results indicate that the mutation in the putative cNLS-like sequence of GP63 does not impair the targeting of GP63 to the nucleus. However, this result does not exclude the possibility for the presence of a non-classical NLS in *Leishmania* GP63, which may be common or specific to *Leishmania* species.

### *Leishmania* Degrades the Nuclear Pore Complex of Host MΦ in a GP63-Dependent Manner

As *Leishmania* GP63 is unlikely to use a cNLS-mediated nuclear import (Fig 2), we investigated other possibility for the protease to achieve nuclear localization. In this context, proteins without cNLS sequences have been shown to trigger nucleocytoplasmic transport through a direct interaction with components of the NPC like Nups [19] and pathogens like viruses have been shown to degrade the NPC and Nups as a way of altering nucleocytoplasmic transport [20]. A sequence screen of Nup proteins for putative GP63 cleavage sites (using the ScanProsite tool), displayed hits for different family members Nup62, Nup358, Nup214 and Nup93 (S1 Dataset).

**Fig 3:**
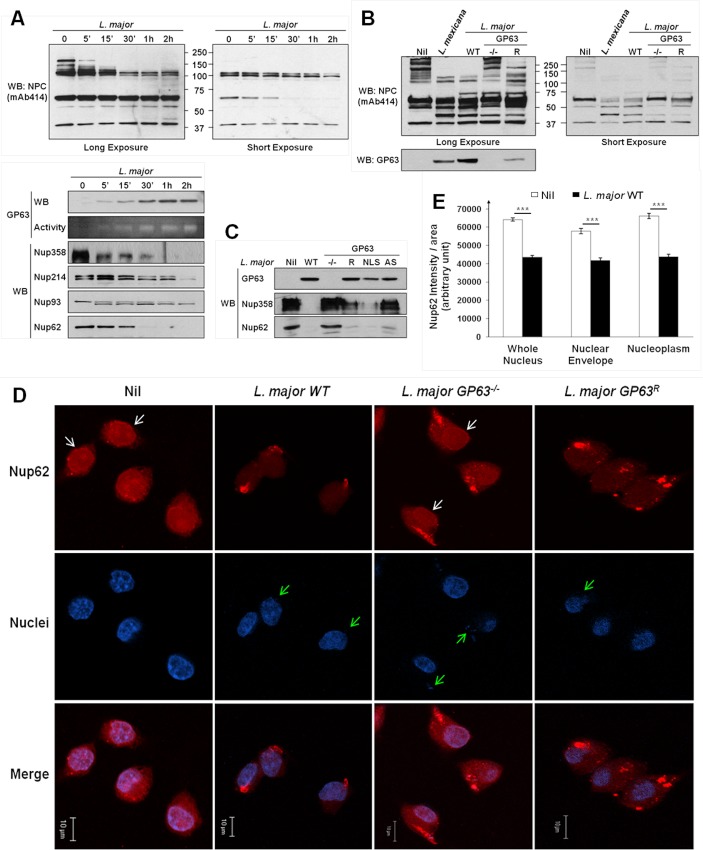
*Leishmania* degrades the NPC of macrophages in a GP63-dependent manner. (A) *L*. *major* degrades NPC Nups. LM1 MΦ cells were infected as indicated with *L*. *major* for different times. Cell lysates were submitted to total protein lysis. Parasite lysate (*Lmaj* Lys) was used as a control. Samples were analyzed either by western blot or by gelatin zymography. An antibody against *Leishmania* GP63 was used to monitor its presence. An antibody against FG-Nups was used to monitor NPC degradation. In addition, antibodies against the FG-Nups Nup358, Nup214, Nup62, and against Nup93 were used. (Also see S1B Fig) (B) NPC degradation is GP63-dependent. LM1 MΦ cells were infected as indicated with either *L*. *mexicana* or *L*. *major* species (WT, GP63^-/-^, GP63^R^) for 2 hrs. Total protein lysates were analyzed by western blot. Parasite lysate (*Lmaj* Lys) was used as a control. A FG-Nup antibody was used to monitor the NPC degradation and a GP63 antibody to monitor its presence. (C) GP63 does not need the NLS but needs to be active to degrade the NPC. LM1 MΦ cells were infected as indicated with *L*. *major* (WT, GP63^-/-^, GP63^R^, GP63^NLS^, GP63^AS^) for 2 hrs. Total protein lysates were analyzed by western blot. Antibodies against Nup358 and Nup62 were used to monitor the NPC degradation and a GP63 antibody to monitor its presence. (D) Confirmation of GP63-dependent Nup62 degradation by confocal microscopy. LM1 MΦ cells were infected as indicated with different species of *L*. *major* (WT, GP63^-/-^, GP63^R^) for 2 hrs. Cells were stained for Nup62. White arrows represent Nup62, green arrows represent parasites. Results are representative of 3 sets of experiments. (E) Quantification of Nup62 after infection with different species of *L*. *major* (WT, GP63^-/-^) in whole nuclei (WN), nuclear envelope (NE), and nucleoplasm (NP). Values are represented +/- SEM. *** p < 1.10^–3^.

Western Blot analysis revealed a degradation of NPC Nup proteins after MΦs were infected either with *L*. *major* WT (Fig 3A), or *L*. *mexicana* (S1A Fig) in a time-dependent manner. Both *Leishmania* species caused cleavage of Nup proteins after infection. Cleavage of Nup62 was also observable after infections with either *L*. *mexicana* or *L*. *major* using only low doses of infection (S2B Fig). This emphasizes the likelihood of a Nup62 cleavage and consequently a NPC-degradation during *in vivo* parasite infections. Interestingly, some Nups were not or weakly affected by *Leishmania* infection, possibly due to a restricted accessibility within the NPC. Moreover, antibodies detecting Nup62 and Nup358 were able to recognize cleavage fragments that were in accordance with the sequence analysis (S2C Fig, S1 Dataset). Indeed, after sequence analysis of Nup62 and Nup358 for GP63 cleavage site using the ScanProsite Tool (S2C Fig), we found an exact and a potential site for GP63-dependent cleavage in the sequence of Nup62 that would result in fragments of ~ 50kDa and ~ 42kDa, as observed in the S2C Fig. In the case of Nup358, six exact cleavage sites for GP63 (and several additional potential ones—S2C Fig) were found and those may explain the cleavage-dependent smears seen in S2C Fig. GP63 cleavage sites were also identified in Nup214 and Nup93.

To investigate whether NPC fragmentation was a result of GP63 activity, we compared infections of MΦs with *L*. *major* WT, GP63^-/-^, GP63^R^ and *L*. *mexicana* parasites (Fig 3B). We observed a substantial degradation of Nups in MΦs infected with *L*. *mexicana*, *L*. *major* WT and GP63^R^, but not with *L*. *major* GP63^-/-^, suggesting a pivotal role of GP63 for NPC degradation. This was further supported by the finding that *L*. *major* GP63^AS^ was not able to target Nups. Proteolytic cleavage of Nups by GP63 was unaffected after infection of MΦs with *L*. *major* GP63^NLS^ parasites (Fig 3C). Confocal microscopy using a Nup62 specific antibody and intensity quantifications confirmed our previous findings (Fig 3D and 3E). Cells infected with *L*. *major* WT and GP63^R^ parasites showed a clear reduction of intensity for Nup62 at the NE and inside the nucleus, while cells infected with *L*. *major* GP63^-/-^ displayed the same cellular Nup62 distribution as non-infected cells (Fig 3D). Nuclear Nup62 intensity was quantified and results for non-infected cells and *L*. *major* WT infected cells were compared. This demonstrated that *L*. *major* infection was responsible for a significant reduction of the Nup62 signal at the NE and inside the nucleus (Fig 3E, S1 Dataset).

The usage of a large array of virulent *Leishmania* species and the non-virulent *L*. *tarentolae* [21] strain in infection experiments also indicated that the GP63-dependent alteration of the NPC, as shown by Nup62 cleavage, is a conserved phenomenon in virulent *Leishmania* species (S2D Fig). Taken together, our data strongly suggests a GP63-dependent alteration of NPCs due to the cleavage of Nups after infection, which may affect the nuclear transport and offer GP63 access to nuclear targets.

### LC-MS/MS Proteomic Analysis of MΦ Nuclei after *Leishmania* Infection

**Fig 4:**
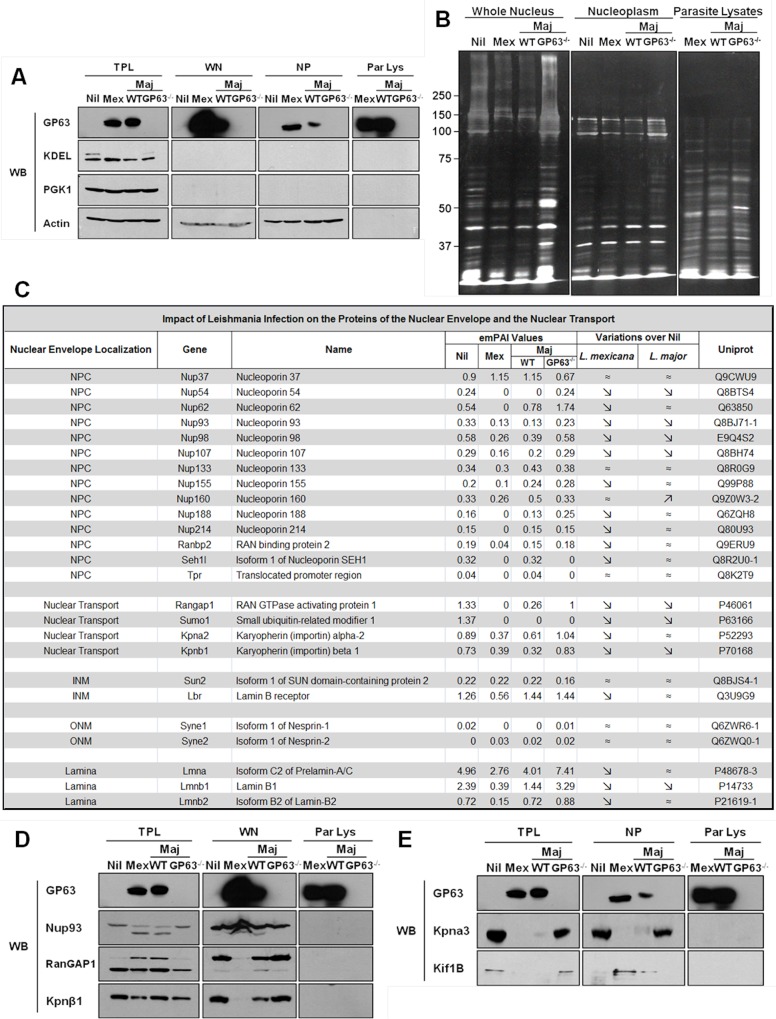
GP63 alters nuclear envelope protein levels and of proteins involved in nuclear transport. (A and B) Analysis of purity of LC-MS/MS samples. (A) LM1 MΦ cells were infected as indicated with *L*. *major* species (WT, GP63^-/-^) or *L*. *mexicana* for 2 hrs. Total protein lysate (TPL), whole nuclei (WN), nuclear envelope (NE), and nucleoplasm (NP) samples were generated. An antibody against GP63 was used to monitor its presence. Antibodies against KDEL (ER marker), PGK1 (cytoplasmic marker), and Actin (loading control) were used as controls. (B) Silver staining of infected samples. LM1 MΦ cells were infected as indicated with *L*. *major* species (WT, GP63^-/-^) or *L*. *mexicana* for 2 hrs. Whole nuclei (WN) and nucleoplasm (NP) lysates were generated. Parasite lysates were used as controls (Par Lys). (C) Impact of *Leishmania* infection on the proteins of the nuclear envelope and the nuclear transport machinery. INM: Inner Nuclear Membrane; ONM: Outer Nuclear Membrane. (D) Confirmation of the LC-MS/MS results for WN samples by western blot. LM1 MΦ cells were infected as indicated with *L*. *major* species (WT, GP63^-/-^) or *L*. *mexicana* for 2 hrs. Total protein lysates (TPL, left panel) and whole nuclei (WN, middle panel) lysates were generated. Parasite lysates were used as controls (Par Lys, right panel). Specific antibodies were used to monitor Nup93, RanGAP1 and Kpnβ1. Antibody against GP63 was used to monitor its presence. (E) Confirmation of the LC-MS/MS results for NP samples by western blot. LM1 MΦ cells were infected as indicated with *L*. *major* species (WT, GP63^-/-^) or *L*. *mexicana* for 2 hrs. Total protein lysates (TPL, left panel) and nucleoplasm (NP, middle panel) lysates were generated. Parasite lysates were used as controls (Par Lys, right panel). Specific antibodies were used to monitor Kpna3 and Kif1B. Antibody against GP63 was used to monitor its presence.

Both previous reports and the preceding results demonstrated that GP63 is able to interfere with the nuclear transport machinery, as well as with the activation of nuclear localized TFs and phosphatases [4,5]. To determine to which extent *Leishmania* and GP63 respectively modify nuclear physiology, whole nuclei (WN) and nucleoplasms (NPs) were purified from murine LM1 MΦs, after infection with *L*. *major* WT, *L*. *major* GP63^-/-^ and *L*. *mexicana* (Fig 4A). The analysis of the protein content of both purified WNs and NPs revealed global differences (Fig 4B), confirming that *Leishmania* infections result in extensive alterations within the nuclei. The protein patterns obtained after *L*. *major* WT and *L*. *mexicana* were similar to each other, while the protein pattern after *L*. *major* GP63^-/-^ infection resembled the results obtained for non-infected cells. These findings support the hypothesis that leishmanial GP63 protease activity plays a crucial role in alterations of nuclear proteins after infection.

Both WNs and NPs were subjected to LC-MS/MS proteomic analysis and the proteomic data analyzed using the exponentially modified protein abundance index (emPAI), which calculates a ratio of observed to observable peptides, based on factors like mass spectrometry analyses sensitivity, biochemical properties of proteins and published empirical data. The emPAI values are proposed to be linearly correlated to protein concentration [22,23].

### GP63 Alters the Nuclear Envelope and Nucleocytoplasmic Transport Machinery

With these criteria, proteomic analysis on WN identified a total of 932 different proteins: 726 in uninfected Nil samples, 684 in *L*. *mexicana*, 694 in *L*. *major* WT, and 762 in *L*. *major* GP63^-/-^ samples (S1 Dataset). Given our previous results, we focused our analysis of WN on proteins from the NE and/or involved in the nucleocytoplasmic transport (Fig 4C). The proteomics data confirmed that protein levels of Nups (including Nup62, Nup93, Nup214 and Nup358, also called RanBP2) were largely decreased or below detection limit in infected cells compared to non-infected cells (Fig 4C). Interestingly, LC-MS/MS proteomic analysis of WN highlighted the fact that other nucleoporins, such as Nup54, Nup98, or Nup107, and proteins involved in nucleocytoplasmic transport (RanGAP1, SUMO1, Importin alpha-2, Importin beta-1) were also altered in dependency of GP63 (Fig 4C). In addition, levels of proteins from the inner nuclear membrane (SUN2), the outer nuclear membrane (Nesprin-1 and 2), and from the lamina (Lmna, Lmnb1 and 2), which are all part of the LINC complex, connecting the nucleoskeleton to the cytoskeleton and involved in mechanotransduction of extracellular stimuli, were also decreased in the presence of GP63 [24].

We validated the LC-MS/MS results for different proteins identified in WN by western blot analysis, confirming the cleavage of Nup93, RanGAP1, and Importin beta-1 (Kpnβ1) in the presence of GP63 (Fig 4D, middle panel). RanGAP1, a Ran GTPase activating protein, which has been implicated in importin-dependent nucleocytoplasmic transport, exists both free in the cytosol (70kDa) and in a sumoylated form, attached to the NPC (90kDa) via Nup358. We discovered that GP63 decreases the protein levels of RanGAP1 at the NE (Fig 4D, middle panel). Consequently, an accumulation of potentially sumoylated RanGAP1 was observable in TPLs after infection and only in the presence of GP63 (Fig 4D, left panel), implying that GP63 may mediate the detachment of the sumoylated RanGAP1 from the NPC and Nup358 specifically [25]. In this regard, it remains unclear whether the detachment depends on Nup358 cleavage. *Leishmania* infections also resulted in diminished Kpnβ1 protein levels in the WN, but only with GP63 present (Fig 4D, middle panel). However, an increase in Kpnβ1 abundance in the cytoplasm was not observable (Fig 4D, left panel). Importin beta-1 facilitates the docking of the Importin alpha/NLS-containing protein (cargo) complex at the cytoplasmic side of the NPC. In the presence of nucleoside triphosphates and the small GTP binding protein Ran, the complex enters the NPC and the importin subunits dissociate. While Importin beta remains at the pore, the complex of Importin alpha/cargo protein is transported through the NP. RanGAP1 increases the rate at which Ran hydrolyzes GTP into GDP in the cytoplasm. A decrease of RanGAP1 and Kpnβ1 at the NE would both be consistent with an impairment of nuclear import.

Moreover, the analysis of the Nups, RanGAP1, and Kpnβ1 proteins sequences revealed the presence of putative GP63 cleavage sites (S1 Dataset). In accordance to the data previously introduced, *L*. *major* GP63^-/-^ induced changes of protein levels were largely negligible (Fig 4C, 4D, and 4E). Thus, our proteomics data further substantiates our hypothesis that *Leishmania* is able to alter NPCs and reveals additional targets of the host nuclear transport machinery that are affected after parasite infection.

### *L*. *major* GP63 Has an Impact on Host MΦ Nucleoplasmic Proteins

For a further characterization of the impact of GP63 on nuclear physiology of host MΦs, nucleoplasms were extracted and submitted to proteomic analysis. Extraction purity and LC-MS/MS results were validated by western blot analysis (Fig 4A and 4E), showing the cleavage of the importin Kpna3 in NP in the presence of GP63 only (Fig 4E, middle panel). Besides the cleavage of proteins involved in nucleocytoplasmic transport we also observed GP63-dependent mislocalization of proteins such as Kif1B (Fig 4E left panel and middle panel) after infection by *L*. *mexicana* and *L*. *major* WT but not after infection by *L*. *major* GP63^-/-^ **(** Fig 4E **)**.

**Fig 5:**
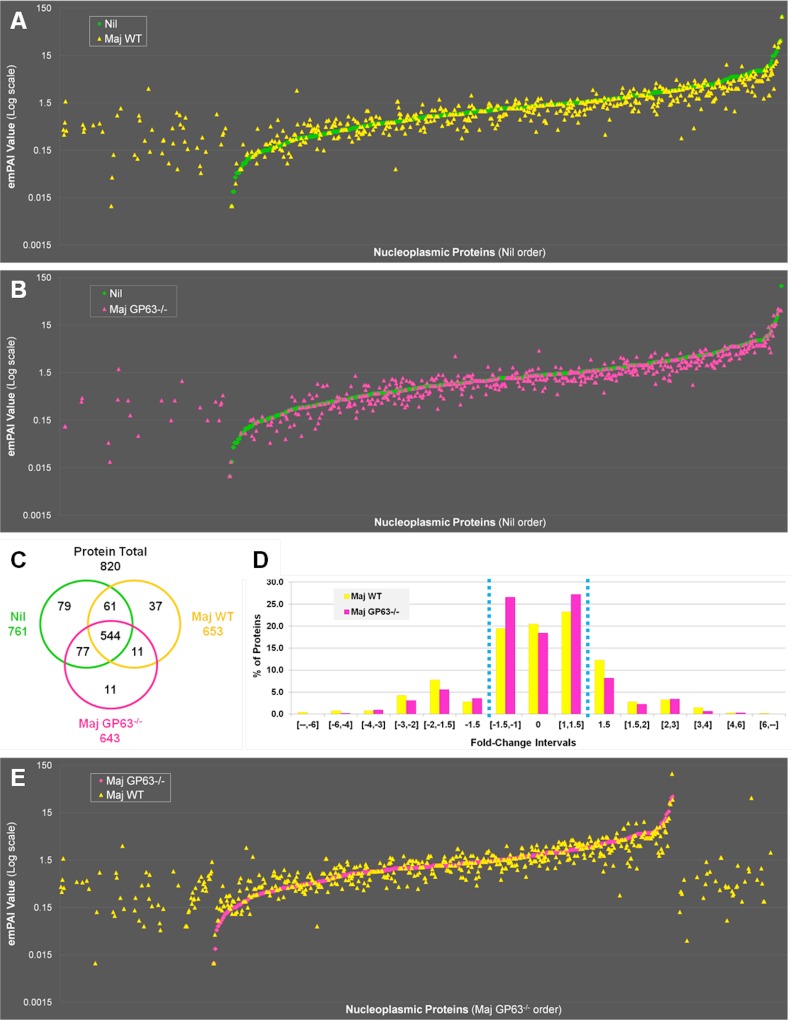
Quantitative proteomic analysis of macrophage nuclei after infection with *L*. *major WT* or *L*. *major* GP63^-/-^. (A) Comparison of nucleoplasmic proteins: *L*. *major* WT samples vs Nil samples. All proteins identified in Nil samples were represented according to their emPAI value (smallest to highest) as green diamonds. All proteins identified in *L*. *major* WT were represented as yellow triangles, according to the protein order of Nil samples. This allows visualizing which proteins are unique, smaller or higher in abundance in the Maj WT samples compared to the Nil ones. (B) Comparison of nucleoplasmic proteins: *L*. *major* GP63^-/-^ samples vs Nil samples. Analysis was carried out as in A with *L*. *major* GP63^-/-^ proteins identified represented as pink triangles. This allows visualizing which proteins are unique, smaller or higher in abundance in the Maj GP63^-/-^ samples compared to the Nil ones. (C) Venn diagram of proteins identified in Nil, *L*. *major* WT and *L*. *major* GP63^-/-^ samples. (D) Analysis of the changes in emPAI values of *L*. *major* WT and *L*. *major* GP63^-/-^ samples. Displayed is the number of proteins (in %) of the *L*. *major* WT samples (yellow) and *L*. *major* GP63^-/-^ samples (pink) according to the fold-change of their emPAI value in comparison to Nil samples. Blue lines correspond to -1.5x and +1.5x fold-change, which were considered as significant values. (E) Comparison of nucleoplasmic proteins: *L*. *major* WT samples vs *L*. *major* GP63^-/-^ samples. All proteins identified in *L*. *major* GP63^-/-^ (and Nil) samples were represented according to their emPAI value (smallest to highest) as pink diamonds. All proteins identified in *L*. *major* WT samples were represented as yellow triangles, according to the *L*. *major* GP63^-/-^ sample protein order. This allows visualizing which proteins are unique, smaller or higher in abundance in the Maj WT samples compared to the Maj GP63^-/-^ ones.

**Fig 6:**
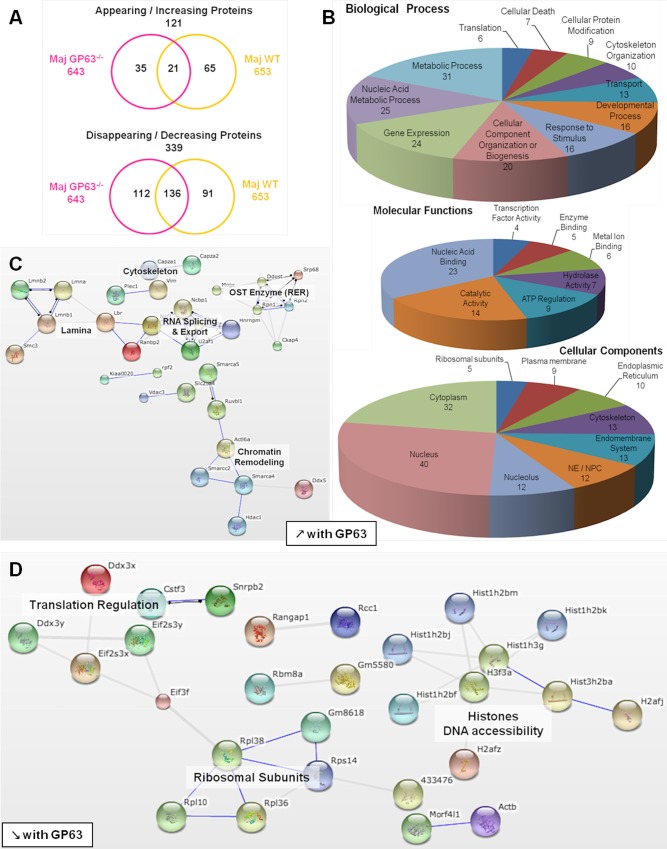
Comparative proteomic analysis of macrophage nuclei after infection with *L*. *major WT* or *L*. *major* GP63^-/-^. (A) Venn diagram of proteins showing increased/decreased levels after *L*. *major* infection (WT or GP63^-/-^) compared to Nil samples. Protein levels were considered significantly increased or decreased at a fold-change ≥1.5X or ≤-1.5X. (B) GO annotations of all proteins with an increased abundance in the presence of GP63. Biological processes, molecular functions and cellular components were displayed. For each section, groups identified as the most represented, and/or the most meaningful, and/or the most interesting concerning our study were selected. Values correspond to the number of proteins and one protein can be part of several groups. (C) String software generated biological network of proteins with an increased abundance in the presence of GP63. String analysis (high confidence—score 0.7) were displayed according to protein function. Only connected nodes were shown. (D) String software generated biological network (as in (c)) of proteins with a decreased abundance in the presence of GP63 (see S3 Fig and S2 Dataset for more).

**Fig 7:**
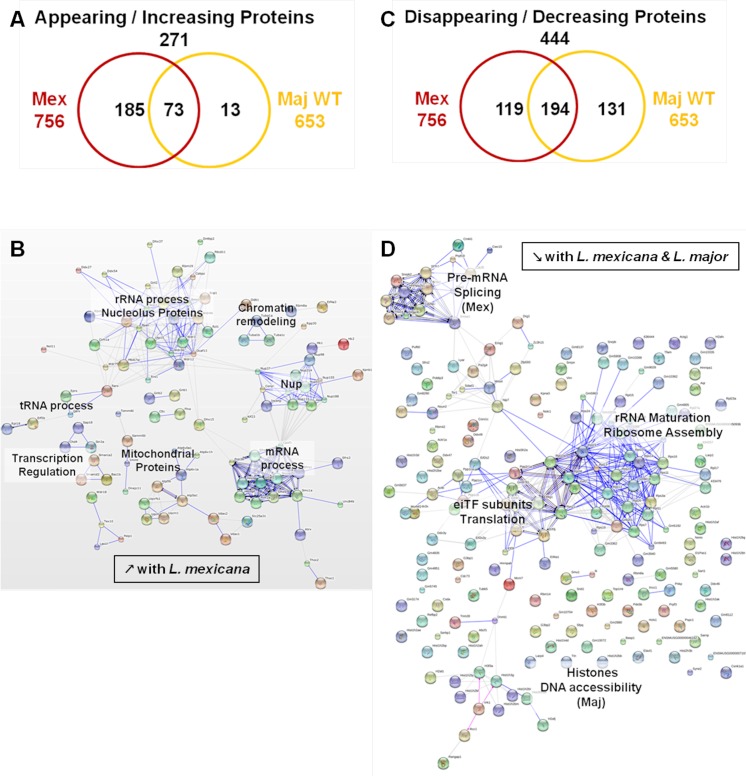
Comparative proteomic analysis of macrophage nuclei after infection with *L*. *major WT* or *L*. *mexicana*. (A) Venn diagram of all proteins with an increased abundance after *L*. *major* WT or *L*. *mexicana* infection compared to Nil samples. Protein levels were considered significantly increased at a fold-change ≥1.5x. (B) String software generated biological network of proteins with an increased abundance after *L*. *mexicana* infection. Results were displayed as an action view from the String software (high confidence—score 0.7). Only connected nodes were shown. (C) Venn diagram of all proteins with a decreased abundance after *L*. *major* WT or *L*. *mexicana* infection compared to Nil samples. Protein levels were considered significantly decreased at a fold-change ≤-1.5X. (D) String software generated biological network (as in (B)) of proteins with a decreased abundance after *L*. *major* WT or *L*. *mexicana* infection (see Figs S4, S5, and S3 Dataset for more).

With the criteria mentioned previously, we identified a total of 996 different proteins by LC-MS/MS analysis: 761 proteins in NP of uninfected cells, 653 proteins after infection with *L*. *major* WT, 643 proteins after *L*. *major* GP63^-/-^ infection and 756 proteins in the case of *L*. *mexicana* infection. We considered a difference in emPAI values significant if the change was at least 1.5 fold, as frequencies analysis demonstrated that the majority of the proteins were under that range (Fig 5D and S4D). For an in-depth analysis of proteins affected by *Leishmania* infection we utilized the STRING-software to generate functional clusters of altered proteins, using gene ontology (GO) annotations. In two separate batches of analyses, we investigated proteins that exhibited changes in abundance in the NP in dependency of GP63 expression (Figs 5 and 6, S3, and S2 Dataset) and compared the differences of the nucleoplasmic protein content after infection with *L*. *major* WT and *L*. *mexicana* (Figs 7, S4, S5, and S3 Dataset).

In order to confirm that GP63 was involved in host nuclear processes, we compared LC-MS/MS results obtained for nuclei of Nil, *L*. *major* WT and *L*. *major* GP63^-/-^ samples (Fig 5). The emPAI values of all the proteins identified for *L*. *major* WT or *L*. *major* GP63^-/-^ samples were displayed in regard to the proteins found in the control sample (n = 820—Nil emPAI values are represented in ascending order) (Fig 5A and 5B). The majority of the proteins identified were detectable in all samples (544). However, depending on the infection status the abundance of individual proteins often differed. Accordingly, our results demonstrate that most alterations after *Leishmania* infection were decreases in protein abundance and at least in part relied on GP63. Furthermore, *L*. *major* WT samples featured more and stronger changes of protein levels than *L*. *major* GP63^-/-^ samples (Fig 5A and 5B). This is also illustrated through the observation that 37 proteins were exclusively detected in *L*. *major* WT samples, while only 11 proteins were unique to L. major GP63^-/-^ samples (Fig 5C). Taken together, 20.5% of proteins detected in non-infected conditions were absent in *L*. *major* WT samples, while 18.4% of proteins were absent in *L*. *major* GP63^-/-^ (Fig 5D). The importance of GP63 was substantiated by direct comparison of nucleoplasmic proteins levels after *L*. *major* WT and *L*. *major* GP63^-/-^ infection of MΦs. *L*. *major* WT infection clearly induced the appearance of a substantial number of proteins and the extent of changes in protein abundance in *L*. *major* WT samples differed markedly (Fig 5E). Thus, the presence of GP63 seems to be a prerequisite for a number of alterations within the NP.

### *L*. *major* GP63 Is Involved in Host Gene Expression and Translation Alterations

Infection with *L*. *major* strains (WT and GP63^-/-^) primarily resulted in a reduction of protein levels in the NP (339). An increase in NP protein levels was less prevalent (121) (Fig 6A).

Among the proteins showing a GP63-dependent increase in abundance (65), 37 proteins were not present at all in NP of uninfected cells (S2 Dataset), again indicating a mislocalization of proteins due to alterations to the NE and the nucleocytoplasmic transport machinery. Using the STRING software and GO annotations, we identified several functional protein clusters which are involved in chromatin remodeling (Actl6a, Smarca4, Smarca5, Smarcc2, Hdac1, Ruvbl1), nuclear RNA splicing and export (Sfrs4, Hnrnpm, U2af1, Ncbp1, Nxf1) and co-translational modification (Ddost, Mogs, Rpn1, Rpn2, Srp68) (Fig 6B and 6C). Interestingly, proteins of the NE as well as proteins of the endoplasmic reticulum and the endomembrane system were found in NP after *L*. *major* WT infection, possibly due to the activity and protein degradation-mediated by GP63 (Fig 6B).

The comparison of *L*. *major* WT and *L*. *major* GP63^-/-^ samples revealed a GP63-dependent decrease of nucleoplasmic protein levels (91) (Fig 6A and 6D, S3, and S2 Dataset). STRING software and GO annotations revealed that a number of NP-proteins, which exhibited a GP63-dependent decrease in abundance, were involved in ribosome assembly (Rpl10, Rpl36, Rpl38) or subunits of the eukaryotic translation initiation factor (Eif2s3x, Eif2s3y, Eif3f). Furthermore, ~40 different histones were identified (Fig 6D).

Although the comparative LC-MS/MS analysis of host nucleoplasms after *L*. *major* WT and *L*. *major* GP63^-/-^ infection identified the protease GP63 as a key factor for the alterations of nuclear physiology, we identified 157 proteins with altered protein levels in both *L*. *major* WT and *L*. *major* GP63^-/-^ samples (Fig 6A). These changes comprised alterations of ATP-dependent mechanisms including enzymatic metabolic processes, as well as nucleic acid metabolism, chromosome organization (helicase activity), gene expression (TF activity / nucleolus, spliceosome) and translation (translation factor activity, ribosome) (S3 Fig, S2 Dataset). Therefore, it is likely that *Leishmania* has GP63-independent means to alter nuclear protein levels as well.

### Comparison of Nucleoplasmic Protein Levels after *L*. *major* and *L*. *mexicana* Infections

In our experiments, we further aimed to elucidate the impact of different *Leishmania* species on the MΦ NP. Thus, we compared infections with *L*. *major* WT and *L*. *mexicana* parasites. We subjected the proteomic data obtained for the *L*. *major* WT and *L*. *mexicana* samples to the same comparative analyses as previously performed for the comparison of *L*. *major* WT and *L*. *major* GP63^-/-^ samples (S4 Fig). The emPAI values of all the proteins identified for *L*. *major* WT and *L*. *mexicana* samples were displayed in consideration of the proteins found in the control sample (n = 920—Nil emPAI values in ascending order) (S4A and S4B Fig). We detected 552 proteins with changed protein levels, which were present in all infected samples. A closer examination of our data revealed that *L*. *mexicana* infection was able to trigger a variety of alterations, which were not present after *L*. *major* infection (S4A and S4B Fig). Indeed, we found 111 proteins whose abundance was only changed after *L*. *mexicana* infection (111/157 proteins), with only two proteins uniquely changed in *L*. *major* WT samples (2/48). (S4C Fig). In addition, it is noteworthy that not only the number of altered proteins is higher in *L*. *mexicana* samples but recorded changes were more extensive (S3D Fig and S4E). This finding possibly reflects the fact that *L*. *mexicana* is considered more virulent than *L*. *major* WT. Therefore, our comparative studies indicate that *L*. *mexicana* parasites alter nuclear physiology to a larger extent than *L*. *major* parasites.

We then performed STRING and GO analyses of the proteins identified in both *L*. *mexicana* and *L*. *major* WT samples (Figs 7, S5, and S3 Dataset). In *L*. *mexicana* samples 258 proteins were detected that either appeared or increased in abundance. This group comprised 85% of the proteins detected in *L*. *major* WT samples (73/86) (Fig 7A). Consequently, we were able to identify similar clusters as in our previous analysis for *L*. *major* WT with new groups unique for *L*. *mexicana* infections (Figs 6B, 6C, and 7B). Thus, the created STRING biological network revealed that both *L*. *mexicana* and *L*. *major* parasites act on chromatin remodeling and transcription regulation. For *L*. *mexicana* samples we identified more detailed clusters distinguishing mRNA, tRNA and rRNA processes (Fig 7B). GO annotations also indicated that nucleic acid metabolic processes were still the predominant functions altered by both *Leishmania* species, but transport activity, signaling and response to stimuli were also strongly affected after *L*. *mexicana* infection (S4 Fig). Newly identified clusters consisted of Nups and mitochondrial proteins (Fig 7C) further substantiating a *Leishmania*-dependent dysregulation of the nucleocytoplasmic transport machinery. Detailed GO annotations confirmed the identification of eight Nups after infection with *L*. *mexicana* possibly due to the effect of GP63 (Figs 2 and 3). The identification of proteins involved in mitochondrial processes within the nucleus after *L*. *mexicana* infection indicates that *L*. *mexicana* may also affect the integrity of other cellular organelles (Figs 7D, S4, and S3 Dataset). The groups of proteins with diminished protein levels after either *L*. *major* WT or *L*. *mexicana* infection were partly overlapping, too. 194 proteins were identified in both *L*. *mexicana* and *L*. *major* WT samples, while 119 were unique to *L*. *mexicana*, and 131 were unique to *L*. *major* WT samples (Fig 7D). STRING and GO analyses highlighted the possibility of a parasite-dependent interference with proteins involved in rRNA maturation and ribosome assembly, as well as translation initiation. However it is interesting to note that *L*. *major* parasites seemed to act mostly on histones, while *L*. *mexicana* parasites impacted strongly on pre-mRNA splicing and consequently mRNA export and translation (Figs 7D, S5, and S3 Dataset).

Together, our data and the observation that GP63 can affect the protein content of host nuclei may offer new explanations for a multitude of observations previously published, including the inhibition of cellular translation and the downregulation of host protective mechanisms, as well as effects on cell proliferation and nucleotide metabolism [5,26,27].

### Analysis of *Leishmania* Proteins within MΦ Nuclei after *L*. *major* and *L*. *mexicana* Infection

Although our study suggests GP63 is of pivotal importance for *Leishmania*-induced changes of nuclear physiology, our results did not exclude the possibility of a direct or indirect dependency on the activity of additional leishmanial proteins. To identify other *Leishmania* factors within the host cell nuclei, we subjected our proteomic samples to the *Leishmania* database. Overall, we detected 94 parasite proteins within host MΦ nuclei. Interestingly, beside GP63, no other known *Leishmania* virulence factors were identified (S1 Dataset), thus substantiating the critical role of GP63 for the alterations of nuclear physiology.

## Discussion

*Leishmania*-mediated subversion of signaling pathways involving protozoan virulence factors has been a subject of high interest in recent years. Although primary indications of *Leishmania*-mediated effects on nuclear physiology existed, no further analysis was carried out to date. For the first time we here present an in-depth proteomic analysis—both quantitative and qualitative—of host MΦ nuclei after *Leishmania* infection with a special focus on the implications of the leishmanial protease GP63. Herein, we provide evidence that the metalloprotease GP63, a key virulence factor of *Leishmania* parasites, can localize to the NE of host MΦs.

With the determinants for GP63 localization within MΦs unknown, we identified a putative cNLS-like sequence within the GP63 sequence of several *Leishmania* species. Classical nuclear import via an NLS sequence is one of the major routes to deliver proteins to the nucleus in higher eukaryotes. Nevertheless, mutation of the cNLS-like sequence did not result in an alteration of GP63-targeting to the NE. Although we have not ruled out the possibility that a non-classical NLS is present in the GP63 sequence, only few reports describe such sequences in protozoan trypanosomatids like *Leishmania* [28,29]. This could support our results that GP63 reaches the NE independently of the identified putative classical NLS sequence, and utilizes a different yet unknown pathway to target the nucleus. A possibility may be an import factor-independent mode of translocation. In this context, some proteins, like β-catenin, have been shown to enter the nucleus via the NPC by an NLS/importin-independent mechanism, where they recognize and bind Nups similar to importin family proteins. These proteins contain specific motifs called Armadillo or HEAT repeats that are recognized by FXFG-motifs of Nups [19,30,31]. A preliminary blast analysis revealed partial matches for Armadillo/HEAT repeats in the amino acid sequence of GP63. In the future, site-directed mutagenesis studies of these motifs could further elucidate whether or not these sequences are determinants of GP63 localization within host cells. It is also possible that the substrate recognition process for Nups itself may play a role in the targeting of GP63 to host MΦ nuclei. In any case the localization of the protease seems to enable an alteration of both nuclear transport proteins and proteins within the nucleoplasm with potentially drastic consequences for key nuclear processes.

Proteomic analysis of *L*. *major* and *L*. *mexicana* infected nuclei finally revealed the extent of parasite-dependent alterations of host nuclear physiology and specifically the impact of GP63. Indeed, after both *L*. *major* and *L*. *mexicana* infection, we observed alterations in the localization and abundance of Nups from the NPC, as well as of different components of the NE, the nucleocytoskeleton, and the nucleocytoplasmic transport machinery. Thus, our proteomic data on WN and NP were in accordance with our results obtained by molecular biology methods. The observed alterations of the NE, the nucleocytoskeleton, and the nucleocytoplasmic transport machinery may very well represent a parasite survival strategy that is a consequence of GP63-passage through the NPC. Although corresponding modification of the nuclear transport machinery and nuclear physiology have not yet been described for any trypanosomatid parasites, our results correlate with studies demonstrating that viruses are able to disrupt the NE or alter the composition and function of the NPC for their own survival and propagation. For instance, the alteration of nucleocytoplasmic transport—including the mislocalization of proteins to the nucleus—has been shown in the case of picornavirus, rhinovirus and poliovirus infections. In these cases the changes have been linked to changes in nucleocytoplasmic transport, signal pathway alterations and an impaired immune response [32]. Interestingly, the mislocalization of proteins to the nucleus and defects in nucleocytoplasmic transport after rhinovirus and poliovirus infections were attributed to the degradation of Nups (Nups 62, 153, 214, and 358), resembling the results we obtained for GP63-dependent cleavage of NPC-proteins [10,33–35]. Here we showed that GP63 seems to act in a similar fashion as proteases from different viruses that have been shown to degrade Nups, including Nup62, to interfere with host nuclear transport and other host nuclear mechanisms.

However, some viruses have been known to utilize interactions with host Nups to their own benefit. For instance, HIV-1 has been shown to specifically use the mislocalization of the host Nup62 to increase HIV-1 gene expression and infectivity [11,36]. During Herpes simplex virus infections, viral ICP27 directly interacts with the nuclear pore complex through Nup62, inhibiting host nucleocytoplasmic transport pathways [37]. Consequently, in this context purified *Leishmania* GP63 could represent an interesting approach for a treatment interfering with these mechanisms of virulence.

The comparison of our proteomic data for *L*. *major* WT and *L*. *major* GP63^-/-^ infection provides further evidence that GP63 is indeed strongly involved in a wide variety of *Leishmania*-induced changes of nuclear physiology besides nucleocytoplasmic transport. This is illustrated by the similarities in our proteomic analysis and comparison of NPs after *L*. *major* WT, *L*. *major* GP63^-/-^ and *L*. *mexicana* infection. Both virulent *Leishmania* species affect whole protein clusters that are of importance for or act in chromatin remodeling, RNA-related processes (transcription, splicing, and export), nucleoskeleton and ribosome maturation. In this regard it is noteworthy that *L*. *major* and *L*. *mexicana* led to a diminished abundance of several subunits forming the eukaryotic initiation Translation Factor (eiTF). This is most likely mediated by GP63 as depicted by the proteomic data obtained after *L*. *major* GP63^-/-^ infection. The finding of GP63 interfering with various eiTF subunits corroborates the results of a previous study. There, *L*. *major* GP63 was shown to inhibit host protein translation through the cleavage of mTOR [26]. Thus, the direct GP63-mediated cleavage of eIF proteins may present a second parasitic strategy to impair cellular translation after infection. Our results also indicated that GP63 is involved in the alteration of proteins from the LINC complex (Sun, Nesprins) and the nuclear lamina (lmna, lmnb1, lmnb2, lmnbr), both crucial for nucleus integrity. Moreover, the LINC complex has also been shown to be involved in the mechanotransduction of extracellular stimuli [24], which may represent yet another novel process for *Leishmania* to evade host protective mechanisms. In addition, lamins and their associated proteins are also involved in other nuclear functions besides the maintenance of the shape and the mechanical strength of the nucleus: chromatin organization, DNA replication, transcription regulation, RNA processing and the linkage of the nucleus to all major cytoskeleton networks [38]. In any case the diversity of the nuclear targets of GP63 substantiates the impact of GP63s’ proteolytic activity on host MΦ function and nuclear physiology.

Our results concerning *L*. *major* WT, *L*. *major* GP63^-/-^ and *L*. *mexicana* infections do not exclude the possibility of additional proteins involved in the nuclear physiology alteration. To our knowledge, a nuclear localization within the host cell after infection has only been proposed for a hypothetical leishmanial protein from *L*. *pifanoi* and *L*. *donovani*. But both protein function and localization could not be confirmed to date [39]. Although our proteomic analysis of nuclei after infection identified various leishmanial proteins that gain access to the nucleus, none of them are parasite virulence factors.

In conclusion we herein show through different methodologies that *Leishmania* GP63 can target the perinuclear region of host cells. At the nuclear envelope the parasite protease is able to alter host nucleoporins, the nucleocytoskeleton, and the nucleocytoplasmic transport through proteolytic cleavage. Moreover, our proteomic data sets greatly increase our understanding how parasites, specifically through GP63, may impact on both host gene expression and translation and our data may explain observations of previous studies in this regard. Furthermore, our data reveals a parasite-mediated overall interference with key processes of nuclei, providing novel leads, as the basis for future functional studies, on how *Leishmania* can potentially subvert host functions to their own benefit. In addition to the cleavage of host transcription factors and the PTP-mediated signaling hijacking, our study strongly suggest that *Leishmania* infections are likely to cause the shutdown of a wide range of integral host cell nuclear and cytoplasmic functions possibly to ensure parasite survival and dampening of anti-leishmanial immune responses. Thus, GP63 arguably represents a possible approach to be included in future research for efficient treatments of *Leishmania* infections as its inhibition could negate the parasites ability to subvert various host-protective functions, not only the dysregulation of protein phosphorylation and inhibition of ROS and NOS production, but also intracellular transport mechanisms, gene expression and translation as presented herein. This is further emphasized by the finding that GP63 can confer resistance against antimicrobial peptides [40,41]. Our findings, and given the importance of GP63 for the subversion of host protective mechanisms early during infection, strengthen approaches that try to introduce either the DNA or the metalloprotease itself in vaccination studies. Generally those approaches have shown elevated success [42–44].

## Materials and Methods

### Cell and Parasite Culture

The immortalized murine bone marrow derived MΦ LM1 cell line (generated in our lab [45]) was maintained in culture at 37°C in 5% CO_2_ in Dulbecco’s Modified Eagle Medium (DMEM) supplemented with 10% heat inactivated FBS and antibiotics (Penicillin 100 U/ml—Streptomycin 100 μg/ml). *Leishmania* promastigotes (*L*. *major* WT, *L*. *major* GP63^-/-^, *L*. *major* GP63^R^, *L*. *major* GP63^NLS^ mutant, *L*. *major* GP63^AS^ mutant, *L*. *mexicana*, *L*. *donovani infantum*, *L*. *donovani donovani*, *L*. *amazonensis*, *L*. *tarentolae*) were grown and maintained at 25°C in SDM-79 culture medium supplemented with 10% heat inactivated FBS. *L*. *major* GP63^R^, *L*. *major* GP63^NLS^ and *L*. *major* GP63^AS^ mutant parasites were selected with geniticin G418 (Sigma-Aldrich, Oakville, ON, Canada, 50 ng/ml). MΦs were infected at a parasite to MΦ ratio of 20:1 with stationary phase promastigotes for the indicated times.

### Total Protein Lysates

To generate total parasite lysates, promastigotes were collected at day 7 by centrifugation, washed 3 times in PBS and lysed with cold buffer (40 mM Tris HCl, 275 mM NaCl, Glycerol 20%, Igepal 1%, 1X protease inhibitor cocktail (PIC); Roche, Mississauga, Ontario, Canada).

To generate cell lysates after infection, LM1 cells were washed 3 times with PBS and submitted to either total protein lysis or nuclear separation. For total protein lysates, cells were lysed with cold lysis buffer (50 mM Tris pH 7.0, 0.1 mM EDTA, 0.1 mM EGTA, 1% Igepal, 0.1% 2-Mercaptoethanol, 1X PIC). Proteins were quantified by Bradford assay (Bio-Rad, Mississauga, Ontario, Canada).

### Whole Nucleus / NE / NP separation

For nuclear separation, we adapted a protocol from Matunis et al. [46]. Washed and recovered LM1 cells were resuspended in cold buffer A (0.25 M sucrose, 25 mM KCl, 5 mM MgCl_2_, 50 mM Tris pH7.5, 1X PIC). After centrifugation (800g, 4°C, 20 min), the cytoplasmic fraction (supernatant) was discarded and the nuclei fraction (pellet) was resuspended in cold buffer A. Subsequently, the nuclei fraction was purified using a sucrose gradient (2M and 1.5M sucrose solutions) by ultracentrifugation (31000 rpm, 4°C, 3 hrs). The isolated nuclei were resuspended in buffer A and counted on hemacytometer (1×10^6^ nuclei = 15μg proteins). To extract NE, whole nuclei were pelleted (5 min, 2500 rpm, 4°C), lysed (500 μl of buffer containing 0.1 mM MgCl2, 1 mM DTT, 5 μg/ml DNaseI, 5 μg/ml RNase A, 1X PIC) and resuspended in 2 ml of extraction buffer pH 8.5 (10% sucrose, 20 mM triethanolamine pH 8.5, 0.1 mM MgCl2, 1 mM DTT, 1X PIC). Nuclei were underlayed with sucrose cushion solution (30% sucrose, 20 mM triethanolamine pH 7.5, 0.1 mM MgCl2, 1 mM DTT, 1X PIC) and NEs were pelleted (4000g, 15 min, 4°C). The NE pellet was resuspended in 500μl of extraction buffer pH 7.5 (10% sucrose, 20 mM triethanolamine pH7.5, 0.1 mM MgCl2, 1 mM DTT, 1X PIC), followed by 50 μl of extraction buffer pH 7.5 containing 0.3 mg/ml Heparin. The NEs were underlayed with sucrose cushion solution, spun (30 min, 4000g, 4°C) and resuspended in extraction buffer pH 7.5.

To extract NP, whole nuclei were pelleted (10 min, 4°C, 5000 rpm), resuspended in buffer C (20 mM HEPES pH 7.9, 0.4 M NaCl, 1 mM EDTA, 1 mM EGTA) and incubated for 20 min at 4°C. NP was collected by centrifugation (13000 rpm, 4°C, 15 min). NE and NP samples were dosed by Bradford assay.

### Western Blots

Protein extracts were treated as previously described [5]. Briefly, between 25 and 40μg of proteins were separated by SDS-PAGE (8%, 10% or 12% acrylamide), and transferred to PVDF membranes. Membranes were blocked in Tris Buffered Saline and tween 0.1% (TBS-T) containing 5% BSA for 1 hr and incubated either 2 hrs at room temperature or over-night at 4°C with primary antibody. After washing with TBS-T (2 times for 5min), membranes were incubated 1 hr with secondary anti-HRP-conjugated antibody (GE Healthcare, Mississauga, ON, Canada). After washing with TBS-T (3 times for 5min), they were developed by chemoluminescence immunodetection with ECL reagents (Thermo Fisher Scientific, Rockford, IL) and autoradiography.

List of antibodies used: GP63 monoclonal antibody clone #253 (Button et al. 1991); Histone H2B (Genscript corporation, A01174); KDEL (ab12223), SHP-1 (ab3254), NPC Mab414 (ab24609), Nup62 (ab96134), Nup358 (RanBP2, ab64276), KpnB1 (ab2811), KpnA3 (ab105348) from Abcam; PGK1 (ProteinTech, 17811-1-AP); Actin (Sigma, A5316); TCPTP (MediMabs, MM-0018-P); NF-κB p65 (SC-8008), Nup93 (SC-374400), Nup214 (SC-26055), RanGAP1 (SC-1862), Kif1B (SC-28540) from Santa Cruz; C-Jun (Cell Signaling, 60A8).

### Gelatin Zymography Assay

Protease activity of GP63 was assayed as previously described by 10% SDS-PAGE incorporated with gelatin (1mg/ml) [47]. With some modifications to the previous protocol, the gels were loaded with parasite extracts (10μg of proteins) that were added to SDS-PAGE sample buffer (15.6mM Tris pH6.8, 2% SDS, 10% glycerol, 0.05% Bromophenol Blue). Electrophoresis was performed at a constant current of 20mA at room temperature. After electrophoresis, SDS was removed by incubation with washing buffer (2.5% Triton X-100 in 50mM Tris pH 7.4, 5mM CaCl_2_, 1μM ZnCl_2_) for 1 hr on a rotating shaker at room temperature. Then, the gels were briefly rinsed twice with deionized water and incubated in a renaturation buffer containing 50mM Tris pH 7.4, 5mM CaCl_2_, 1μM ZnCl_2_, over-night at 37°C. After incubation, gels were stained 30 min in 0.5% Coomassie Brilliant Blue R-250 in 30% ethanol and 10% acetic acid, and destained for several hours in a solution containing 30% ethanol and 10% acetic acid. Active GP63 was detected as clear bands on the gel.

### PTP In Gel Assay

In-gel PTP assay was performed as previously described [3]. Briefly, poly(Glu,Tyr) substrate was tyrosine-phosphorylated by overnight (O/N) incubation with GST-FER protein kinase (10μg) and 150 μCi [γ-^32^P]dATP. The substrate was then incorporated in a 10% SDS-polyacrylamide gel at a concentration of 2×10^5^ cpm/ml. Mϕ protein extracts, prepared as described above, were denatured for SDS-PAGE and loaded onto the gel. After electrophoresis, the gel was incubated O/N in the fixative buffer A (50mM Tris-HCl pH 8.0, 20% isopropanol), washed twice 30 min with buffer B (50mM Tris-HCl pH 8.0, 0.3% β-ME), and followed by full protein denaturation in buffer B containing 6M guanidine hydrochloride and 1mM EDTA. Gels were washed twice 1 hr in buffer C (50mM Tris-HCl pH 7.4, 1mM EDTA, 0.3% β-ME, and 0.04% Tween 20) and incubated for final renaturation O/N in Buffer C. Gels were dried and exposed to x-ray film. Active PTPs were detected as clear bands on the film.

### Confocal Microscopy

The day before the infection, cells were plated in 24 well-plates on poly-L-lysine coated and UV sterilized coverslips. After 2 hrs of infection, cells were fixed, stained with DAPI to visualize nuclei, and anti-Nup62 antibody to detect the nucleoporin, and the fluorescence was quantified (65 non-infected cells vs. 36 infected cells—S1 Dataset) as previously described [48]. In parallel, as a control, *Leishmania*-infected macrophages were stained with anti-GP63 antibody to show the perinuclear localization of GP63 within the cells, as described previously [5].

### Mass Spectrometry Analysis

#### Protein digestion with trypsin

A standard TCA protein precipitation was first performed to remove detergents from the samples. Protein extracts were then re-solubilized in 10 μL of a 6M urea buffer. Proteins were reduced by adding 2.5 μL of the reduction buffer (45 mM DTT, 100 mM ammonium bicarbonate) for 30 min at 37°C, and then alkylated by adding 2.5 μL of the alkylation buffer (100 mM iodoacetamide, 100 mM ammonium bicarbonate) for 20 min at 24°C in the dark. Prior to trypsin digestion, 20 μL of water was added to reduce the urea concentration to 2M. 10 μL of the trypsin solution (5 ng/μL of trypsin sequencing grade from Promega, 50 mM ammonium bicarbonate) was added to each sample. Protein digestion was performed at 37°C for 18 hrs and stopped with 5 μL of 5% formic acid. Protein digests were dried in a vacuum centrifuge and stored at -20°C until LC-MS/MS analysis.

### LC-MS/MS, Protein Identification and Bioinformatic Analyses of Proteomic Data

LC-MS/MS analysis was carried out as described before [49]. Protein database searching was performed with Mascot 2.2 (Matrix Science) against the NCBI *Mus musculus* and *Leishmania* protein databases. The mass tolerances for precursor and fragment ions were set to 10 ppm and 0.6 Da, respectively. Trypsin was used as the enzyme allowing for up to 2 missed cleavages. Carbamidomethyl and oxidation of methionine were allowed as variable modifications.

Duplicates of separately analyzed sets of MS/MS data were used for calculation of the exponentially modified Protein Abundance Index (emPAI) values using emPAICalc web server (http://empai.iab.keio.ac.jp/). Mascot output files were uploaded to emPAICalc server and hits with a minimum of 3 peptides and a minimum score of 20 were chosen as true hits for further analyses. Gene Ontology (GO) annotations of identified proteins were extracted and protein-protein interaction networks of the identified proteins were created using STRING database with specific parameters (action view, high confidence—score 0.7) [50].

## Supporting Information

S1 Fig Visualization of GP63 perinuclear localization by confocal microscopy. MΦ cells were infected by *L*. *major*, as described previously. Cells were labeled with an anti-GP63 antibody to visualize distribution of GP63 in infected cells and DAPI was used to stain cell nuclei. The arrow points toward an internalized parasite.
(TIF)

S2 Fig Nucleoporin degradation by other *Leishmania* species. (A) LM1 MΦ were infected with *L*. *mexicana* for different times and total protein lysates were analyzed by western blot to monitor GP63, NPC degradation and more particularly Nup358 and Nup62. (B) Dose-dependent cleavage of Nup62. LM1 MΦ were infected for 2 hrs with the *Leishmania* species indicated. Total protein lysates were analyzed using western blot and specific antibodies for either Nup62 or GP63. Actin was used as a loading control. (C) Potential cleavage profile for Nup62 and Nup358 by *L*. *major* WT. (D) Nup62 is degraded by other *Leishmania* species. Left panel: *L*. *mexicana*, *L*. *infantum*, *L*. *donovani*, *L*. *amazonensis*, and *L*. *tarentolae* parasites were tested for GP63 activity and presence. Right panel: LM1 MΦ were infected with the different *Leishmania* species and Nup62 degradation was monitored in the presence of GP63. Actin was used as a loading control.
(TIF)

S3 Fig GO annotations for proteins altered in the host macrophage nucleoplasm after *L*. *major* WT and GP63^-/-^ infections. Biological processes, molecular functions and cellular components are shown. For each part, only the most represented groups among the nucleoplasmic proteins found in our samples are shown. For some of them, several small groups serving the same process or the same function have been pooled together to be more representative (For more details about groups see: http://amigo.geneontology.org/cgi-bin/amigo/amigo?session_id=3364amigo1373393964). Bars represent the number of proteins. One protein can be part of several groups. Sample Maj WT is represented with yellow bars and sample Maj GP63^-/-^ with pink bars.
(TIF)

S4 Fig Quantitative proteomic analysis of host macrophage nuclei infected or not (Nil) by *L*. *major WT* (Maj WT) or *L*. *mexicana* (Mex). (A) Comparison of nucleoplasmic proteins: Maj WT proteins vs Nil proteins. All the proteins identified in Nil are represented according to their emPAI value (smallest to highest) in green diamonds. All the proteins identified in Maj WT are represented in yellow triangles, according to the Nil protein order. This allows visualizing which proteins are unique, smaller or higher in abundance in the Maj WT samples compared to the Nil ones. (B) Comparison of nucleoplasmic proteins: Mex proteins vs Nil proteins. Same as in (A) with Mex proteins identified represented with red triangles. This allows visualizing which proteins are unique, smaller or higher in abundance in the Mex samples compared to the Nil ones. (C) Venn diagram of the proteins identified in Nil, Maj WT and Mex samples. (D) Analysis of the changes in emPAI values of Maj WT and Mex samples. Displayed is the number of proteins (in %) of the Maj WT samples (yellow) and Mex samples (red) according to the fold-change of their emPAI value in comparison to Nil samples. Blue lines correspond to -1.5X and +1.5X fold-change, considered as the significant values in our study. (E) Comparison of nucleoplasmic proteins: Maj WT proteins vs Mex proteins. All the proteins identified in Maj WT samples (and Nil samples) are represented according to their emPAI value (smallest to highest) in yellow diamonds. All the proteins identified in Mex samples are represented in red triangles, according to the Maj WT sample protein order. This allows visualizing which proteins are unique, smaller or higher in abundance in the Mex samples compared to the Maj WT ones.
(TIF)

S5 Fig GO annotations for proteins altered in the host macrophage nucleoplasm after *L*. *major* and *L*. *mexicana* infections. Biological processes, molecular functions and cellular components are shown. For each part, only the most represented groups among the nucleoplasmic proteins found in our samples are shown. For some of them, several small groups serving the same process or the same function have been pooled together to be more representative (For more details about groups see: http://amigo.geneontology.org/cgi-bin/amigo/amigo?session_id=3364amigo1373393964). Bars represent the number of proteins. One protein can be part of several groups. Sample Maj WT is represented with yellow bars and sample Mex with red bars.
(TIF)

S1 Dataset Global proteomics and statistics data for all the proteins identified and used in all the analyses. Excel file. Sheet 1: *Leishmania* proteins detected in host macrophage nuclei. Sheet 2: emPAI values for all proteins. Sheet 3: Microscopy data for Nup62. Sheet 4: Scaffold number of spectra set 1. Sheet 5: Spectrum report set 1. Sheet 6: Peptide report set 1. Sheet 7: Scaffold number of spectra set 2. Sheet 8: Spectrum report set 2. Sheet 9: Peptide report set 2. Sheet 10: Fold-change of emPAI values after *Leishmania* infection in NP compare to Nil. Sheet 11: *Leishmania* database—proteomic data.
(XLSX)

S2 Dataset Proteins altered in the host macrophage nucleoplasm after *L*. *major* WT and GP63^-/-^ infections. Excel file. Sheet 1: Proteins increasing/appearing after infection. Sheet 2: Proteins decreasing/disappearing after infection.
(XLSX)

S3 Dataset Proteins altered in the host macrophage nucleoplasm after *L*. *major* and *L*. *mexicana* infections. Excel file. Sheet 1: Proteins increasing/appearing after infection. Sheet 2: Proteins decreasing/disappearing after infection.
(XLSX)
