# Supplementary figures and images for: Impact of Leishmania Infection on Host Macrophage Nuclear Physiology and Nucleopore Complex Integrity
Source: PLoS Pathog. 2015 Mar 31;11(3):e1004776. doi: 10.1371/journal.ppat.1004776 (PMC4380401; doi:10.1371/journal.ppat.1004776)

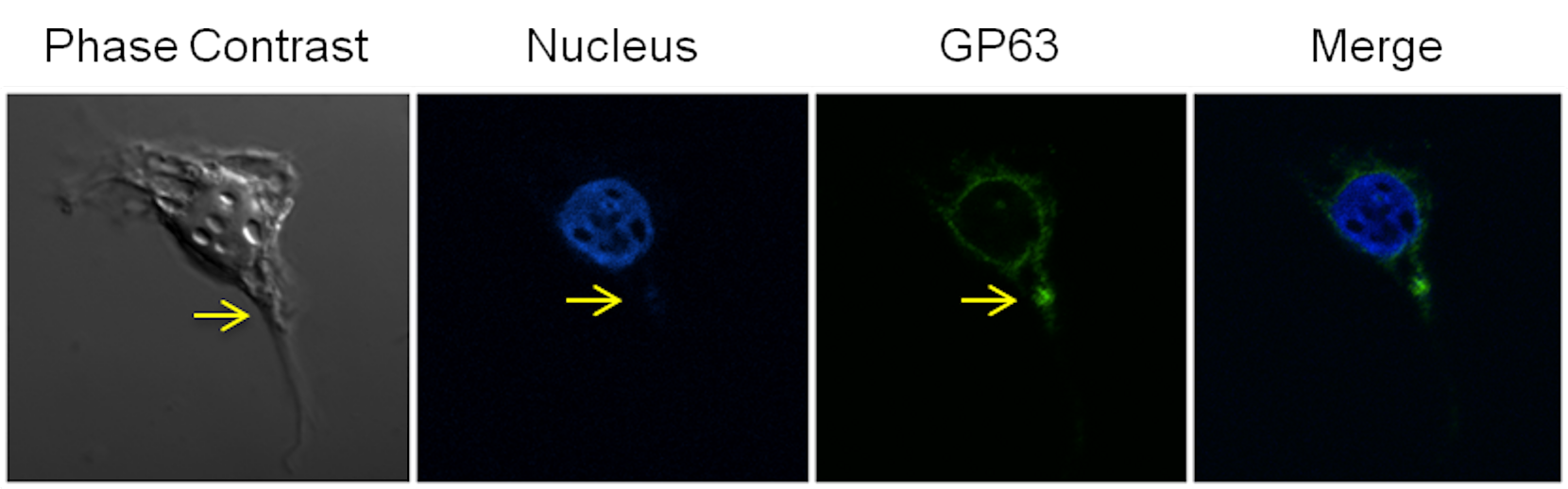

Supplement: S1 Fig — MΦ cells were infected by L. major, as described previously. Cells were labeled with an anti-GP63 antibody to visualize distribution of GP63 in infected cells and DAPI was used to stain cell nuclei. The arrow points toward an internalized parasite. (TIF) [file ppat.1004776.s001.tif]

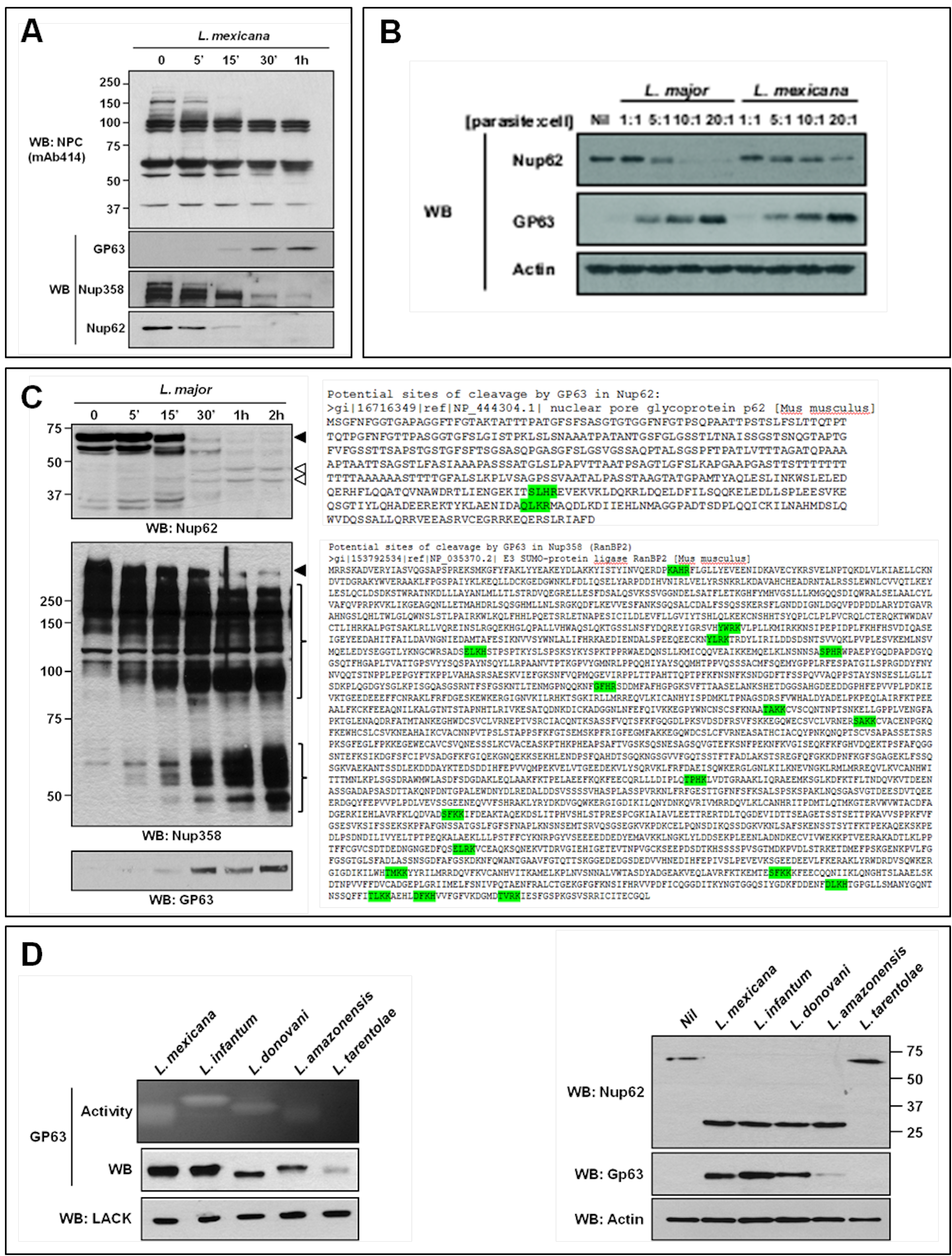

Supplement: S2 Fig — (A) LM1 MΦ were infected with L. mexicana for different times and total protein lysates were analyzed by western blot to monitor GP63, NPC degradation and more particularly Nup358 and Nup62. (B) Dose-dependent cleavage of Nup62. LM1 MΦ were infected for 2 hrs with the Leishmania species indicated. Total protein lysates were analyzed using western blot and specific antibodies for either Nup62 or GP63. Actin was used as a loading control. (C) Potential cleavage profile for Nup62 and Nup358 by L. major WT. (D) Nup62 is degraded by other Leishmania species. Left panel: L. mexicana, L. infantum, L. donovani, L. amazonensis, and L. tarentolae parasites were tested for GP63 activity and presence. Right panel: LM1 MΦ were infected with the different Leishmania species and Nup62 degradation was monitored in the presence of GP63. Actin was used as a loading control. (TIF) [file ppat.1004776.s002.tif]

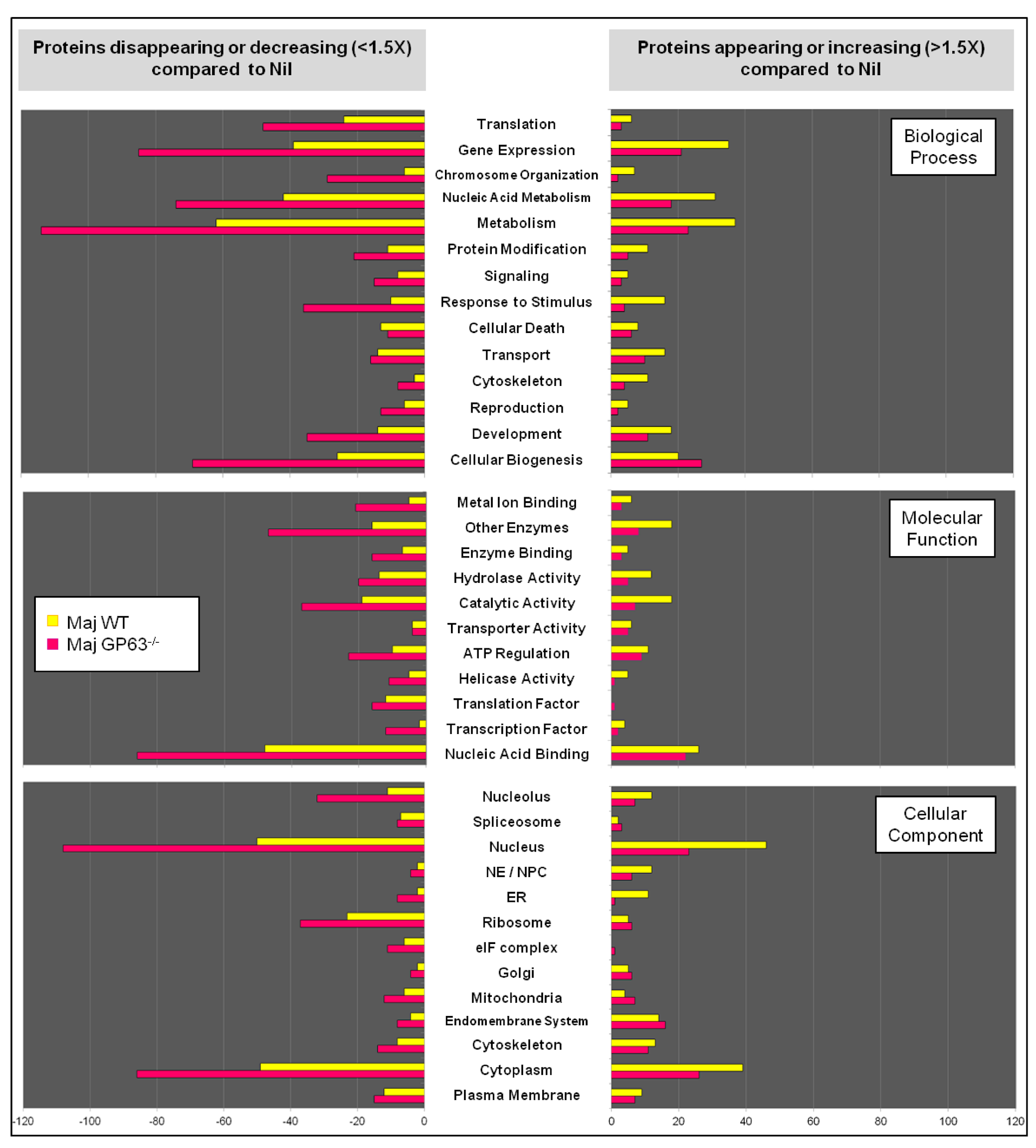

Supplement: S3 Fig — Biological processes, molecular functions and cellular components are shown. For each part, only the most represented groups among the nucleoplasmic proteins found in our samples are shown. For some of them, several small groups serving the same process or the same function have been pooled together to be more representative (For more details about groups see: http://amigo.geneontology.org/cgi-bin/amigo/amigo?session_id=3364amigo1373393964). Bars represent the number of proteins. One protein can be part of several groups. Sample Maj WT is represented with yellow bars and sample Maj GP63-/- with pink bars. (TIF) [file ppat.1004776.s003.tif]

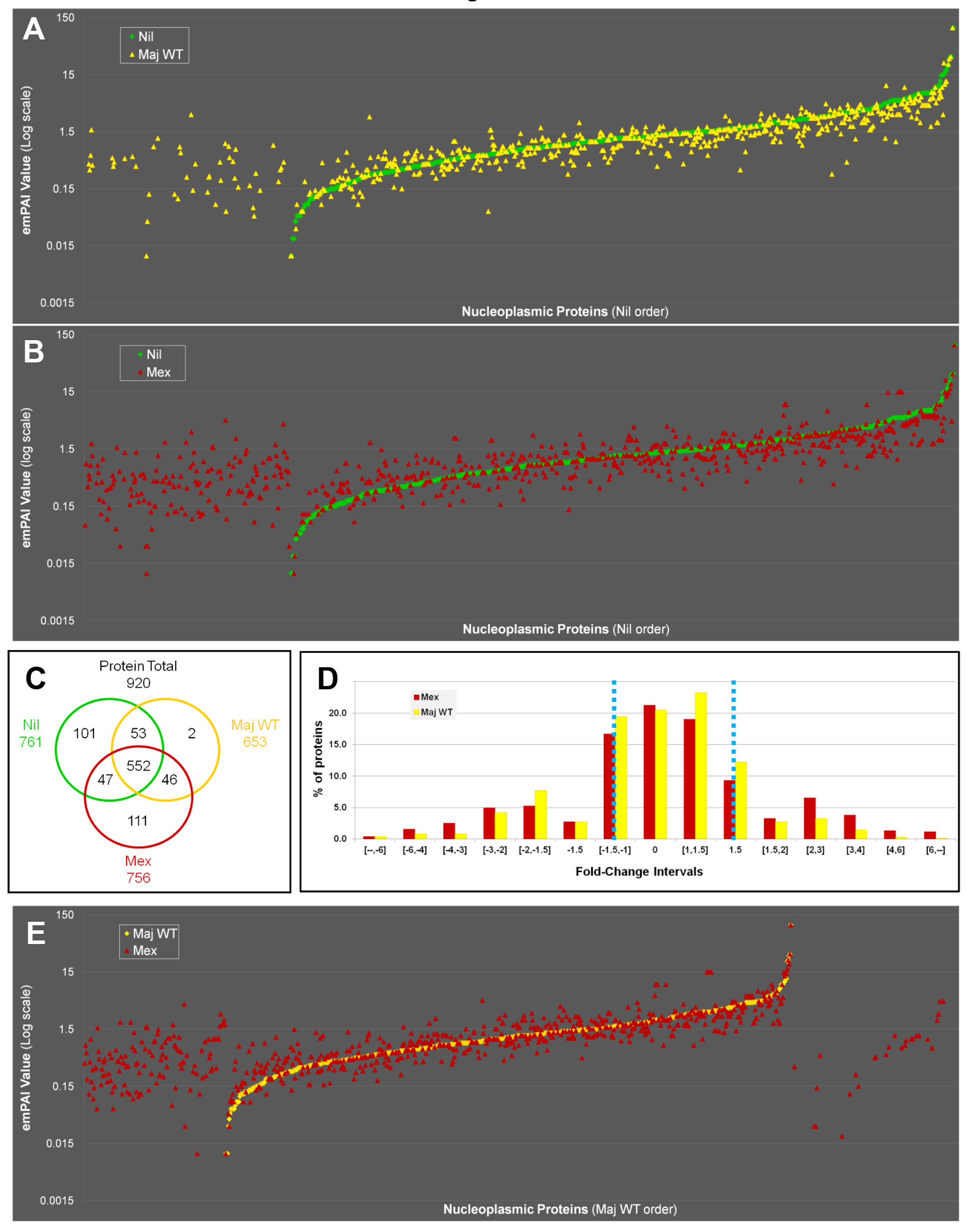

Supplement: S4 Fig — (A) Comparison of nucleoplasmic proteins: Maj WT proteins vs Nil proteins. All the proteins identified in Nil are represented according to their emPAI value (smallest to highest) in green diamonds. All the proteins identified in Maj WT are represented in yellow triangles, according to the Nil protein order. This allows visualizing which proteins are unique, smaller or higher in abundance in the Maj WT samples compared to the Nil ones. (B) Comparison of nucleoplasmic proteins: Mex proteins vs Nil proteins. Same as in (A) with Mex proteins identified represented with red triangles. This allows visualizing which proteins are unique, smaller or higher in abundance in the Mex samples compared to the Nil ones. (C) Venn diagram of the proteins identified in Nil, Maj WT and Mex samples. (D) Analysis of the changes in emPAI values of Maj WT and Mex samples. Displayed is the number of proteins (in %) of the Maj WT samples (yellow) and Mex samples (red) according to the fold-change of their emPAI value in comparison to Nil samples. Blue lines correspond to -1.5X and +1.5X fold-change, considered as the significant values in our study. (E) Comparison of nucleoplasmic proteins: Maj WT proteins vs Mex proteins. All the proteins identified in Maj WT samples (and Nil samples) are represented according to their emPAI value (smallest to highest) in yellow diamonds. All the proteins identified in Mex samples are represented in red triangles, according to the Maj WT sample protein order. This allows visualizing which proteins are unique, smaller or higher in abundance in the Mex samples compared to the Maj WT ones. (TIF) [file ppat.1004776.s004.tif]

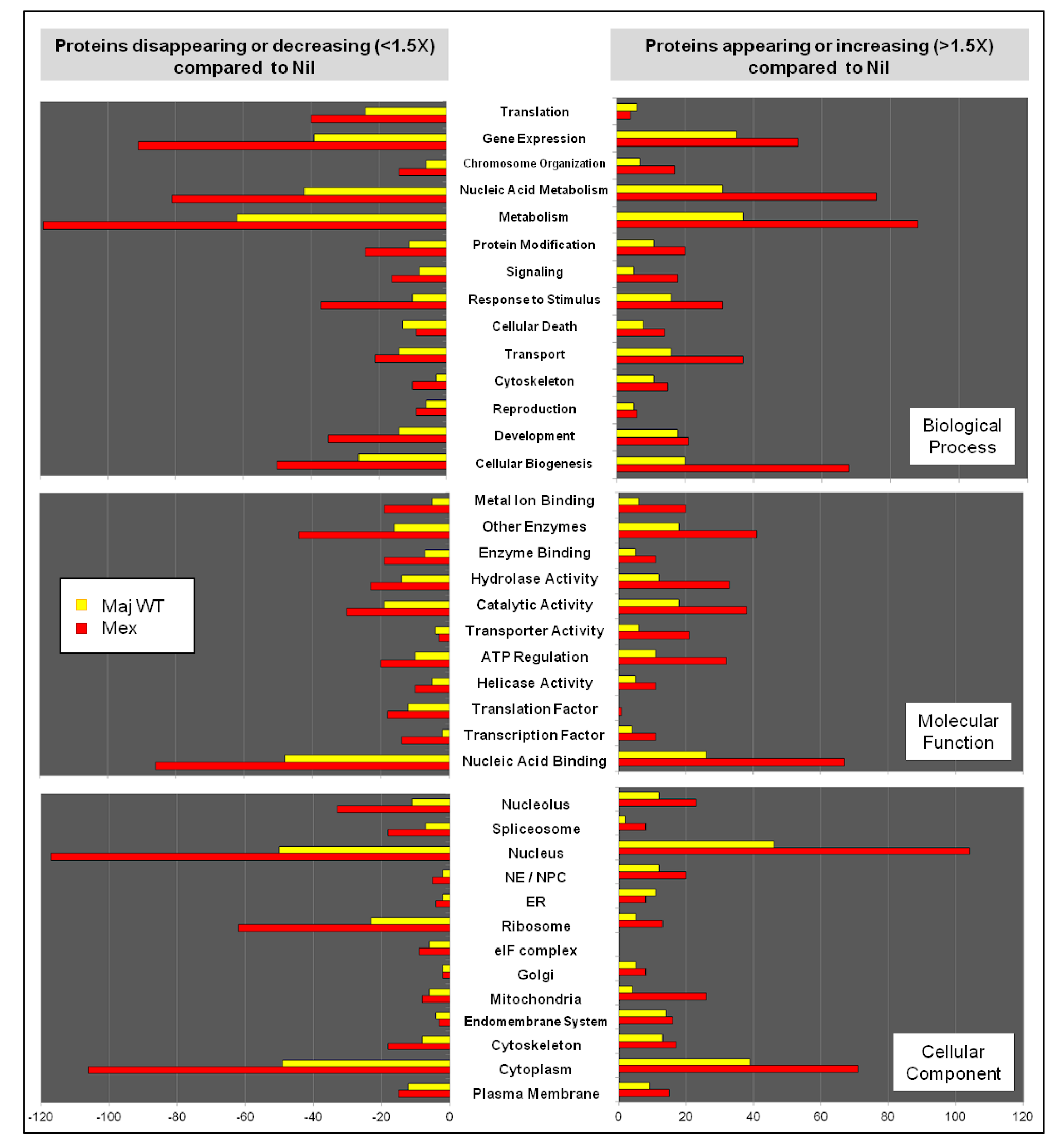

Supplement: S5 Fig — Biological processes, molecular functions and cellular components are shown. For each part, only the most represented groups among the nucleoplasmic proteins found in our samples are shown. For some of them, several small groups serving the same process or the same function have been pooled together to be more representative (For more details about groups see: http://amigo.geneontology.org/cgi-bin/amigo/amigo?session_id=3364amigo1373393964). Bars represent the number of proteins. One protein can be part of several groups. Sample Maj WT is represented with yellow bars and sample Mex with red bars. (TIF) [file ppat.1004776.s005.tif]
